# *Cyberlindnera jadinii* (teleomorph *Candida utilis*) candidaemia in a patient with aplastic anaemia: a case report

**DOI:** 10.1099/jmmcr.0.005160

**Published:** 2018-07-10

**Authors:** Pauline Treguier, Marion David, Gilles Gargala, Vincent Camus, Aspasia Stamatoullas, Anne-Lise Menard, Pascal Lenain, Nathalie Contentin, Emilie Lemasle, Helene Lanic, Hervé Tilly, Fabrice Jardin, Stéphane Lepretre

**Affiliations:** ^1^​ INSERM U1245, Department of Hematology, Centre Henri Becquerel, Université of Rouen Normandie, Rouen, France; ^2^​ Laboratory of Microbiology, Centre Henri Becquerel, Rouen, France; ^3^​ Laboratory of Parasitology-Mycology, CHU de Rouen, Rouen, France

**Keywords:** candidaemia, *Cyberlindnera jadinii*, medullary aplasia

## Abstract

**Introduction.:**

We present what is believed to be the first report of candidaemia caused by *Cyberlindnera* (*Pichia*) *jadinii* (teleomorph of *Candida utilis*) in a patient with an aplastic anaemia.

**Case presentation.:**

The patient, a 21-year-old male, presented with hepatic cytolysis, cutaneous and pulmonary involvement, and septic shock. *Cyberlindnera jadinii* was identified by aerobic blood culture and MS. The patient initially received multiple and combined antifungal therapy, but continued to have persistent skin lesions and fever. He was successfully treated by emergency haploidentical haematopoietic stem cell transplantation, combined with triple antifungal therapy and supportive care.

**Conclusion.:**

*Cyberlindnera jadinii*, teleomorph of *Candida utilis*, which is not usually invasive, can lead to an opportunistic invasive infection in unhealthy adult patients. For treatment of the invasive candida infection, it is necessary to combine antifungal therapy and supportive care.

## Introduction

Herein, we describe what is to the best of our knowledge the first report of candidaemia caused by *Cyberlindnera* (*Pichia*) *jadinii* (teleomorph of *Candida utilis*) in a patient with an aplastic anaemia.

## Case report

A 21-year-old male was admitted to the Centre Henri Becquerel, Rouen, France, with asthenia, fever (39 °C) and icterus on July 15 2016. The patient reported having had these symptoms for 2 weeks. Laboratory investigations indicated pancytopenia (polynuclear neutrophils=0.6 g l^−1^; platelets=6 g l^−1^; haemoglobin=12.5 g dl^−1^), acute cytolytic and cholestatic hepatitis, and inflammatory syndrome. Viral serology was negative. A myelogram revealed low bone marrow density and an absence of megakaryocytes. Medullary biopsy confirmed medullary aplasia without fibrosis. The karyotype was normal, and the patient was negative for paroxysmal nocturnal haemoglobinuria clones and Fanconi disease. The patient, who worked in car body repair, had no relevant medical history. In the absence of another aetiology, a diagnosis of aplastic anaemia was established.

The same day, empirical antibiotherapy with piperacillin/tazobactam (4 g/0.5 g every 8 h) i.v. was initiated. A physical examination on July 21 2016 did not reveal the source of the fever, and abdominal ultrasound and echocardiography were normal. Six blood cultures were taken between July 14 and 25 2016. Two (July 23 and 25) were positive for yeast. *Cyberlindnera jadinii* was identified from aerobic cultures by MS. (The MICs are shown in Table 1).

**Table 1.** Susceptibility data for *Cyberlindnera jadinii*

Drug	MIC at 48 h (mg l^−1^)	Susceptible (S)/intermediate (I)/resistant (R)
Fluconazole	4	I
Voriconazole	0.032	S
Amphotericin B	0.047	S
Anidulafungin	0.002	S
Caspofungin	0.032	S
5-Fluorocytosine	0.008	S

Caspofungin therapy was initiated on July 26 2016 (70 mg on day 1, followed by 50 mg day^−1^). The same day, the patient presented a diffuse, purplish maculo-papular eruption. Cutaneous fungal infection was suspected, but the cutaneous biopsy was inconclusive. On July 29 2016, the patient displayed persistent fever despite the caspofungin therapy, so caspofungin was replaced by intravenous (i.v.) liposomal amphotericin B (3 mg kg^−1^ day^−1^).

On August 1 2016, the patient still presented with a continuous fever (40 °C), and the cutaneous eruption was extensive. Pulmonary computed tomography showed alveolar and nodular opacities with fuzzy contours evoking a fungal infection. Due to the patient’s worsening condition and the computed tomography results, combined therapy with caspofungin (50 mg every day i.v.) and i.v. liposomal amphotericin B (3 mg kg^−1^ every day) was decided. The patient continued to be febrile until August 4 2016, so tazocillin was replaced by imipenem (1000 mg every 8 h i.v.) and trimethoprim/sulfamethoxazole (2400 mg/480 mg every day) to treat uncertain pulmonary pneumocystosis. A new cutaneous biopsy was performed and, after 9 days of culture, *Malassezia* sp. was found. A non-pathogenic species was suspected.

Bronchoscopy on August 8 2016 did not reveal mucosal lesions. However, macrophages and amphotericin B-sensitive *Candida kefyr* were found in the bronchoalveolar lavage while the patient was receiving combined antifungal therapy.

Because of persisting skin lesions and fever, on August 9 2016, a new cutaneous biopsy was performed, revealing an inflammatory perivascular reaction of the dermis and a remodelling of hypodermic fat with mononuclear mobilization. The patient was transferred to an intensive care unit on August 11 2016 for septic shock and probable invasive candidaemia. While there, the patient received i.v. noradrenaline for a few hours. The patient became hypothermic, so external warming was performed. Because of the patient’s prolonged neutropenia, vancomycin (2 g day^−1^ i.v.) was introduced. Because his clinical condition improved, vancomycin was stopped on August 25 2016. The patient continued to have cockade-like skin lesions and no evidence of viral aetiology, with negative PCR for cytomegalovirus, parvovirus B19, Epstein-Barr virus and herpes simplex virus.

The patient was transferred back to the Centre Henri Becquerel on August 15 2016. He continued to present night fever, and the cutaneous eruption gradually improved.

*Aspergillus* galactomannan antigenaemia was consistently negative. In agreement with the *Cyberlindnera jadinii*-positive blood culture, positivity to β-d-glucan (146 pg ml^−1^; negative <60 pg ml^−1^), assessed September 5 2016, confirmed a fungal infection.

To manage the aplastic anaemia, haematopoietic stem cell transplantation (HSCT) was considered. While awaiting a compatible donor, the patient received classical doses of granulocyte-colony stimulating factor and erythropoietin to treat the anaemia. The patient’s 24-year-old brother and other related donors, however, were not HLA-identical. Although the patient did not meet the criteria for HSCT in aplastic anaemia (age <40 years and no HLA-identical familial donor) [1], given the severity of the patient's illness and the absence of a related or unrelated HLA-identical donor, haploidentical HSCT using bone marrow from the patient’s brother was indicated. The patient was A negative and his brother was O negative. Both the patient and his brother were positive for Epstein-Barr virus, and the patient was immunized for cytomegalovirus.

**Fig. 1.**
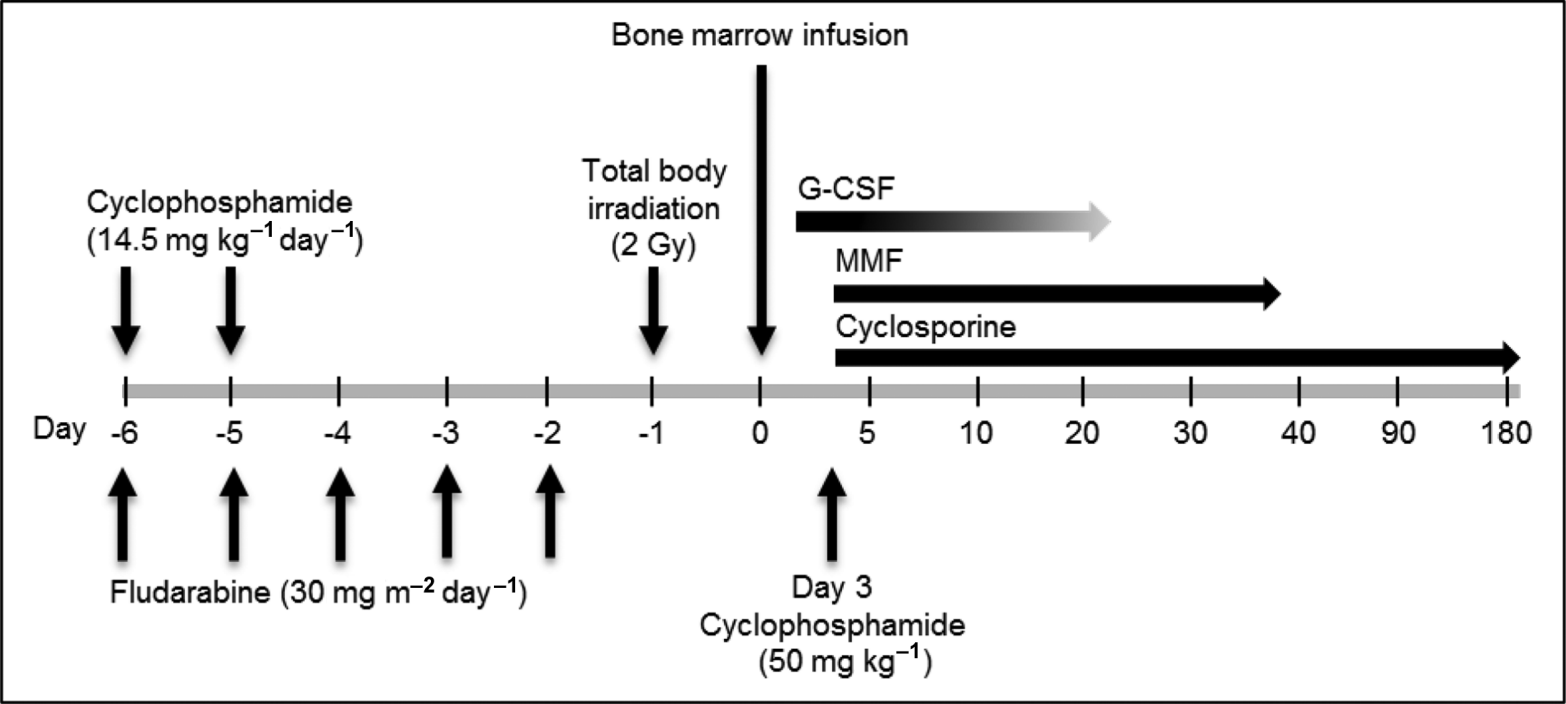
Non-myeloablative conditioning and prophylaxis for GVHD. The patient received non-myeloablative conditioning including cyclophosphamide and fludarabine starting on day −6 and total body irradiation on day −1. Therapy for GVHD included granulocyte-colony stimulating factor (G-CSF), mycophenolate mofetil (MMF) and cyclosporine.

On September 9 2016, 6 days before peripheral stem cell infusion, a non-myeloablative conditioning regimen and prophylaxis for graft-versus-host disease (GVHD) were initiated (Fig. 1). Prior to the transplant, the patient was clinically stable with only temporary (12 h) fever. Bone marrow transplantation was performed on September 15 2016.

No immediate toxicity of the conditioning regimen or graft was found. On day 1, the patient remained febrile with a temperature of ≥39 °C. Thoracic computed tomography revealed nodular opacities evoking a mucosal infection. Voriconazole (4 mg/kg evey day i.v.) was added to the continued antifungal therapy with caspofungin and liposomal amphotericin B. Bronchoscopy and bronchoalveolar lavage were negative.

The patient became afebrile on day 8. Voriconazole was stopped 48 h later because the patient tested negative for aspergillus, and all antibiotics were progressively terminated. Caspofungin was replaced by fluconazole (200 mg every day i.v.) on day 22. Starting on day 19, the patient received oral cyclosporine (2 mg/kg evry day i.v.) to prevent GVHD, and on day 27, the patient received valganciclovir due to cytomegalovirus reactivation.

The last platelet transfusion was performed on day 16 and the last blood transfusion on day 17. On day 18, the patient was no longer aplastic, and on day 26, platelets were >20 g l^−1^. The patient did not display acute GVHD. The whole blood chimerism was 100 % 7 months after the transplantation.

One-year post-transplantation, the patient decided to stop all treatments. In January 2018, the patient was in complete remission without GVHD.

## Discussion

This report describes what is believed to be the first case of *Cyberlindnera* (*Pichia*) *jadinii* (*Candida utilis*) candidaemia in a patient with aplastic anaemia. *Cyberlindnera jadinii* is reported to be the teleomorphic parental species for *Candida utilis*, a non-pathogenic yeast used as a food additive [2]. *Candida utilis* is reported to been opportunistic pathogen and has been isolated from a few superficial clinical specimens taken from immunocompromised patients [3], newborns [4] and elderly adults [5]. Only a few cases of *Candida utilis* candidaemia have been described. The first was in 1988 in a 5-year-old male haemophiliac with AIDS and a central catheter [6]. We suspect that this yeast entered the patient’s bloodstream after colonizing his digestive tract. Indeed, the fungal infection did not come from the central catheter. The patient had peripheral catheters up to the allogenic HSCT, which were changed every 48 h, and peripheral catheter cultures were never positive for *C. jadinii.* In addition, the delay between the positivity of the blood cultures for *C. jadinii*, and the clinical presentation, fever and cutaneous eruption, was concordant.

According to the treatment algorithm for aplastic anaemia [1], patients <40 years of age without a matching donor should be first treated with horse anti-thymocyte globulin and cyclosporine. In addition, invasive fungal infections have been historically considered a contraindication for allogeneic HSCT [7, 8]. Nevertheless, allogeneic HSCT was performed because of the patient’s persistent fungal infection and fever despite triple antifungal therapy. Furthermore, recent retrospective data indicate that allogeneic HSCT is feasible in patients with invasive fungal infections [7, 8].

This case highlights the importance of invasive candida infection and the possibility that *Candida utilis*, not usually invasive, can lead to an opportunistic invasive disease even in otherwise healthy adult patients. In such cases, early diagnosis of the fungal invasion and adapted supportive care are essential.
